# Supervisory psychological support and graduate student creativity: a serial mediation model

**DOI:** 10.3389/fpsyg.2026.1857534

**Published:** 2026-08-12

**Authors:** Xia Tao, Shang Xu, Ruihua Li

**Affiliations:** 1 College of Marxism, Wuhan Institute of Technology, Wuhan, China; 2 Faculty of Basic Education, Putian University, Putian, China

**Keywords:** graduate student creativity, leader-member exchange (LMX), self-determination theory, supervisory psychological support, team innovation climate

## Abstract

Extant research has examined the link between supervisory support and graduate student creativity (PGC), yet the underlying psychological and social mechanisms remain underexplored. This cross-sectional study, grounded in self-determination theory (SDT), examined a serial mediation model in which supervisory psychological support is positively associated with PGC through a sequential two-stage statistical pathway: first via its association with the quality of leader–member exchange (LMX), and subsequently via a positive team innovation climate (TIC). We surveyed 363 full-time graduate students from mainland Chinese universities and examined the hypothesized model using bootstrap resampling with 10,000 iterations. LMX and TIC jointly accounted for the majority of the association between supervisory psychological support (SPS) and PGC, with LMX also serving as a significant mediator in the relationship between SPS and TIC. Our findings suggest that in the high-power-distance, collectivist Chinese academic context, the association between supervisory support and creativity is predominantly accounted for by relational quality: LMX appears to serve as a critical relational mechanism between psychological need satisfaction and creative output. These findings extend the application of SDT to hierarchical academic mentoring contexts and highlight the culturally contingent nature of SDT’s predictions. The results also provide evidence-based guidance for optimizing supervisory training and team innovation management in collectivist higher education settings. Because this study was cross-sectional, the proposed pathways should be interpreted as theory-guided statistical associations rather than evidence of causal effects or temporal ordering.

## 1 Introduction

Graduate student creativity is a core driver of scientific and technological advancement (Meng et al., 2017; Zhou and Shalley, 2003), and supervisors serve as a pivotal factor in students’ creative development. While extant research has examined the relationships between leadership styles and student creativity (e.g., Ma et al., 2023; Gu et al., 2015), three critical research gaps persist in the literature. First, self-determination theory (SDT) posits that creativity is fostered when individuals’ basic psychological needs are satisfied (Ryan and Deci, 2017), yet the specific pathway through which supervisors link supportive behaviors to creative outcomes remains unclear. Relational quality has been proposed as a potential mediating mechanism in this process (Chen et al., 2009). Second, prior research has predominantly focused on Western educational contexts, neglecting how cultural characteristics—specifically high power distance and collectivism—may alter the support–creativity linkage (Ouyang et al., 2025). Third, existing studies have largely framed psychological support as a direct predictor of creativity, while overlooking the potential mediating role of perceived team innovation climate in transmitting supervisory support into creative outcomes. To fill this research gap, this study integrates self-determination theory (SDT) and leader–member exchange (LMX) theory to examine how supervisory psychological support is associated with graduate student creativity through relational and environmental pathways. Specifically, we examined the serial mediation pattern through which supervisory psychological support is statistically linked to creative outcomes via high-quality LMX relationships and team innovation climate (TIC).

Supervisor support is crucial to students’ growth (Miao et al., 2025). Self-determination theory (SDT) posits that support can satisfy three basic psychological needs: autonomy, competence, and relatedness (Ryan and Deci, 2017). This satisfaction is linked to learning motivation and contributes to students’ development. However, in academic contexts, supportive behaviors are not automatically associated with greater creativity. To facilitate student creativity, supervisors must move beyond merely providing task-related advice and develop a nuanced understanding of students’ academic circumstances and research ideas (Guay et al., 2008). Beyond the content of support itself, the relational climate fostered by supportive behaviors also shapes the effectiveness of support (Jang et al., 2010). Unlike traditional support focused on problem solving or emotional comfort, psychological support grounded in self-determination theory aims to address the three basic needs of autonomy, competence, and sense of belonging, thus providing a deeper and more lasting motivation for creative output (Ryan and Deci, 2017). This environment itself has a nourishing effect, continuously provides a psychological sense of security, and, through daily interaction, is conducive to students’ exploration and creative behavior (Appleton et al., 2008). Accordingly, this study raises the first research question: in the Chinese academic context, how is supervisors’ psychological support, grounded in self-determination theory, associated with graduate student creativity?

Studies have shown that supervisors’ psychological support is associated with students’ basic psychological need satisfaction, intrinsic motivation, and creative engagement (Ryan and Deci, 2017; Guay et al., 2008), and is thus related to the development of creativity. However, how and under what conditions such support is associated with creativity—especially in situations of interpersonal interaction and cooperation in academic guidance—has not been fully explained. The supervisor–student relationship is a key factor in determining academic success in higher education (Mainhard et al., 2009). Because the association between psychological support and creativity largely depends on the quality of interaction (Liden and Maslyn, 1998), this study draws on leader–member exchange (LMX) theory to explain the mechanism connecting mentor support with creative outcomes. Unlike traditional leadership theory, LMX focuses on the dual relationship between leaders and members (Graen and Uhl-Bien, 1995), emphasizing that high-quality relationships characterized by trust, reciprocity, and emotional connection (Volmer et al., 2012) can provide key resources and a sense of psychological security, which is conducive to innovative behavior (Meng et al., 2017). In academic situations, the supportive behavior of supervisors (such as autonomy support, competence support, and belonging support) is expected to facilitate high-quality LMX relationships (Liden et al., 1997; Scandura and Pellegrini, 2008). We hypothesize that systematic psychological support can cultivate such relationships, which in turn are expected to be associated with students’ creative achievements. Based on this theoretical logic, this study raises the second research question: do the quality of leader–member exchange and perceived team innovation atmosphere serve as sequential mediators in the association between supervisors’ psychological support and the creativity of graduate students?

Given that the mentor–student relationship is embedded in a broader team and organizational context, this association may be shaped by team-level factors. Team innovation climate (TIC) is defined as the common perception shared by team members regarding the extent to which the team supports innovation (Anderson and West, 1998), through which high-quality teacher–student relationships may relate to innovative outcomes via resource integration and risk buffering (Hülsheger et al., 2009). Accordingly, this study proposes that LMX and team innovation climate mediate the association between supervisors’ psychological support and students’ creativity. At the same time, this study examines whether LMX mediates the association between psychological support and team innovation climate, thus revealing the associational “relationship–environment” pattern underlying creativity.

Integrating self-determination theory (SDT) and leader–member exchange theory (LMX) in the cultural background of high power distance, this study offers three specific contributions. Theoretically, this study explains how psychological needs are expected to be met through high-quality exchange relations to facilitate social transmission in relation to creativity, thus expanding the applicability of self-determination theory (SDT) in hierarchical mentoring contexts. Methodologically, this study uses a chain mediation framework to clarify the “relationship–environment” mechanism, suggesting that leader–member exchange plays a necessary social transformer role (Chen et al., 2009), rather than merely an intermediary variable. In practice, the results of this study provide an evidence-based intervention plan for optimizing mentor training and team innovation management in the collectivist education system.

In summary, by integrating the theory of self-determination with the theory of leader–member exchange, this study constructs a chain mediation model to systematically examine how supervisors’ psychological support is associated with the creativity of graduate students. It is expected that the research results will advance the understanding of the innovative path in academic guidance and offer implications for the design of efficient mentor guidance and team-level intervention programs in higher education.

## 2 Materials and methods

### 2.1 Research design and participants

This cross-sectional quantitative study employed structural equation modeling to test the proposed serial mediation model. The study was conducted among full-time graduate students from multiple comprehensive and research-intensive universities in mainland China. Given the professional nature of the participant population and the need to ensure sufficient variance in participants’ perceptions of supervisory psychological support, this study employed a purposive sampling strategy across multiple universities and disciplines to maximize sample diversity (Palinkas et al., 2015). This method has been widely used in methodological and pedagogical research targeting specific professional groups.

An *a priori* power analysis using G*Power (Faul et al., 2009) indicated that a minimum sample size of 160 was required to detect a small-to-medium effect (f^2^ = 0.15) in multiple regression with four predictors, assuming *α* = 0.05 and statistical power = 0.80. To account for potential invalid responses and attrition, we aimed to collect approximately 400 questionnaires. A total of 400 questionnaires were distributed through a professional online questionnaire platform. After data-quality screening, 363 valid responses remained, meaning that 37 responses (9.25% of distributed questionnaires) were excluded. Because a single questionnaire could violate more than one screening rule simultaneously, exclusion counts are reported as an aggregate total rather than as mutually exclusive categories. This approach avoids artificially inflating the precision of data-quality screening. Two criteria were used to identify invalid responses: (a) missing data exceeding 10% of key items, and (b) straight-lining (identical responses across all Likert-scale items). The 10% threshold was chosen to reduce bias from incomplete data without discarding marginally usable cases; straight-lining was taken as an indicator of inattentive or careless responding. Both criteria follow established recommendations for cleaning online survey data (Meade and Craig, 2012; DeSimone et al., 2015). The final valid sample was *N* = 363, reflecting an effective response rate of 90.75%, which was higher than the standard threshold of online research (Nulty, 2008). Prior to data collection, all potential participants were presented with an electronic informed consent statement on the survey platform. This statement clearly outlined the study purpose (i.e., exploring the relationship between supervisors’ psychological support and graduate student creativity), the anonymous and voluntary nature of participation, the confidentiality of individual responses, participants’ right to withdraw at any time without penalty, and the estimated completion time of 8 to 12 min. Only participants who voluntarily clicked “agree to participate” could proceed to the questionnaire items. This study was conducted in accordance with the ethical principles for research involving human subjects as outlined by the American Psychological Association (APA) and the Declaration of Helsinki. Given the anonymous, non-invasive, and survey-based nature of this research, formal institutional ethical review was not required.

Table 1 presents detailed demographic characteristics of participants and descriptive statistics for the core study variables. The sample demonstrated a relatively balanced gender distribution and was predominantly composed of junior graduate students, which is consistent with prior evidence that early-stage graduate students are more likely to participate in survey research.

**Table 1:** Demographic characteristics of participants (*N* = 363).

Sample characteristics	Category	Frequency	Percentage (%)
Gender	Male	173	47.7
	Female	190	52.3
Age	21–25 years	276	76.0
	26–30 years	83	22.9
	30+	4	1.1
Grade	First-year	159	43.8
	Second-year	177	48.8
	Third-year	27	7.4
Discipline	Science and Engineering	144	39.7
	Humanities and Social Sciences	219	60.3

### 2.2 Theoretical framework

#### 2.2.1 Self-determination theory: psychological needs and creativity

Self-determination theory (SDT) posits that human flourishing and optimal functioning—including creative expression—are facilitated when individuals experience satisfaction of three innate psychological needs: autonomy (the need to experience choice and volition in one’s actions), competence (the need to feel effective and capable in one’s pursuits), and relatedness (the need to feel connected to and cared for by others; Ryan and Deci, 2017). In educational contexts, need satisfaction is hypothesized to foster intrinsic motivation, deep cognitive engagement, and willingness to explore novel ideas, all of which are proximal antecedents of creativity (Deci and Ryan, 2000). When students perceive that their learning environment supports these needs, they are more likely to internalize the value of creative activities, persist in the face of obstacles, and generate original solutions (Guay et al., 2008).

Within the graduate supervision context, supervisory psychological support (SPS) represents a contextual resource through which these three needs may be satisfied. Autonomy-supportive behaviors—such as offering meaningful choice in research directions and acknowledging students’ perspectives—are hypothesized to be associated with greater self-directed exploration. Competence-supportive feedback—such as scaffolding difficult tasks and providing informational rather than controlling evaluations—is hypothesized to be associated with enhanced confidence in one’s creative capabilities. Relatedness-supportive actions—such as expressing warmth, respect, and unconditional regard—are hypothesized to be associated with a secure base from which students feel safe to take creative risks (Allen et al., 2022). Although prior research has often examined these needs in isolation, SDT emphasizes that the simultaneous satisfaction of all three needs produces synergistic effects on motivation and well-being (Ryan and Deci, 2017). Supervisory psychological support, by systematically targeting all three needs within the mentoring relationship, is therefore hypothesized to represent a particularly potent predictor of graduate student creativity. Accordingly, we propose:

*H1*: Higher supervisory psychological support is positively associated with higher graduate student creativity (SPS → PGC).

Beyond its direct association with creativity, SDT further suggests that need-supportive behaviors function as relational catalysts. When supervisors satisfy students’ basic psychological needs—particularly relatedness and competence—students are more likely to experience a high-quality relationship characterized by trust and mutual commitment (La Guardia et al., 2000). From the perspective of leader-member exchange (LMX) theory, such need-supportive behaviors serve as the foundation for developing mature partnerships based on reciprocal trust and obligation (Graen and Uhl-Bien, 1995). In turn, students are expected to reciprocate through heightened engagement and openness, thereby deepening the relational bond. This reciprocity dynamic aligns with SDT’s contention that need satisfaction not only energizes individual motivation but also constructs high-quality interpersonal exchanges (Graen and Cashman, 1975; Blau, 1986). Therefore, supervisory psychological support is expected to cultivate high-quality leader–member exchange (LMX) relationships:

*H2*: Higher supervisory psychological support is positively associated with higher leader–member exchange quality (SPS → LMX).

Finally, SDT-based support behaviors may also function as ambient stimuli that shape students’ perceptions of the broader team environment. When supervisors systematically provide autonomy encouragement, competence feedback, and emotional regard, these behaviors transmit implicit signals about the team’s norms and priorities (Eisenberger et al., 2002; Schneider and Reichers, 1983). Students may generalize from individualized supervisory support to infer that the team as a whole encourages exploration, tolerates failure, and values innovation. This inference process is consistent with social information processing perspectives positing that individuals form perceptions of organizational characteristics partly through interpersonal cues provided by direct leaders (Kozlowski and Doherty, 1989). Thus, we hypothesize:

*H3*: Higher supervisory psychological support is positively associated with higher perceived team innovation climate (SPS → TIC).

#### 2.2.2 Leader-member exchange theory: relational quality as a social converter

Leader–member exchange (LMX) theory conceptualizes supervisor–subordinate relationships as varying systematically in quality, ranging from high-quality exchanges characterized by mutual trust, respect, and reciprocal obligation (the “in-group”) to low-quality exchanges limited to formal, contractual role obligations (the “out-group”) (Graen and Uhl-Bien, 1995). In academic mentoring contexts, high-quality LMX is reflected in supervisors’ willingness to share resources, provide personalized guidance, and offer socio-emotional support beyond formal instructional duties, whereas low-quality LMX is confined to bureaucratic, transactional interactions with minimal personalized investment (Liden and Graen, 1980; Scandura and Pellegrini, 2008).

High-quality LMX is hypothesized to be associated with graduate student creativity through two primary mechanisms. First, it provides resource access and psychological safety. Students in high-quality LMX relationships are expected to receive preferential access to cutting-edge knowledge, academic networks, and instrumental guidance—resources that constitute the material foundation for innovation. Simultaneously, the mutual trust inherent in high-quality LMX is expected to reduce students’ fear of failure, thereby encouraging non-traditional thinking and risk-taking behaviors (Edmondson, 1999; Newman et al., 2017). Second, high-quality LMX is expected to facilitate intrinsic motivational processes. The emotional connection and sense of being valued that characterize high-quality exchanges are hypothesized to enhance students’ willingness to invest effort in creative tasks and to persist when encountering setbacks (Meng et al., 2017). Accordingly, we propose:

*H5*: Higher leader–member exchange quality is positively associated with higher graduate student creativity (LMX → PGC).

Furthermore, LMX is hypothesized to operate as a “relationship lens” through which students interpret supervisory behaviors and perceive their surrounding environment (Vidyarthi et al., 2010). Social cognitive theory posits that individuals actively interpret social cues in their environment, and these interpretations shape subsequent behaviors (Bandura, 1986). In academic contexts, students’ interpretation of supervisory support is expected to be systematically filtered through the quality of the LMX relationship. Students in high-quality LMX relationships—characterized by trust and emotional connection (Liden and Maslyn, 1998)—are expected to generalize their individualized support perceptions to the broader team level, perceiving the team as innovation-supportive. This generalization process transforms individualized support into team-level signals, which actively shapes students’ perceptions of the team innovation climate (Aarons et al., 2014). Conversely, students in low-quality LMX relationships, lacking relational depth and psychological safety, are expected to frame supportive behaviors as isolated or transactional, and are therefore less likely to perceive a positive team innovation climate (Edmondson, 1999; Vähämäki et al., 2021). Thus, LMX represents the core pathway through which relational quality translates into perceived team innovation climate:

*H4*: Higher leader–member exchange quality is positively associated with higher perceived team innovation climate (LMX → TIC).

#### 2.2.3 Team innovation climate and the serial transmission mechanism

Team innovation climate (TIC) refers to the shared perception among team members that the team’s norms, policies, and practices encourage and support innovation (Anderson and West, 1998; Newman et al., 2020). Although originally conceptualized as a team-level construct, following the psychological climate literature (James and James, 1989), the present study operationalizes TIC at the individual level as perceived team innovation climate—specifically, graduate students’ subjective evaluation of the extent to which their research team supports creative endeavors. This individual-level perception is hypothesized to influence creativity through three psychological mechanisms: (1) risk tolerance, the belief that the team provides psychological safety for experimentation and does not impose punitive consequences for failure (Edmondson, 1999; Frazier et al., 2017; Carmeli and Gittell, 2009); (2) resource coordination, the perception that the team facilitates the integration of diverse knowledge, perspectives, and interdisciplinary methods (West, 2002); and (3) goal consensus, the shared recognition that innovation is a core strategic priority of the team (West, 2002).

From a resource-based perspective, a strong innovation climate is expected to facilitate the utilization of vertical resources obtained through high-quality LMX (e.g., mentor guidance, priority access to opportunities) by promoting their integration with horizontal resources available within the team (e.g., peer knowledge, collaborative support). According to conservation of resources theory, this integration is expected to produce synergistic effects, improving the efficiency with which relational resources are converted into innovative outcomes (Chen and Hou, 2016; Hülsheger et al., 2009). In terms of risk buffering, a perceived innovation climate that is inclusive of failure is expected to reduce students’ anxiety about potential negative consequences (e.g., blame, loss of face), thereby encouraging exploratory activities such as seeking feedback, sharing unconventional views, and iteratively refining ideas (Newman et al., 2017). Such climates cultivate the belief that failure is an inherent part of the learning process (Edmondson and Lei, 2014), further reinforcing students’ willingness to experiment without fear of negative evaluation. Therefore, we hypothesize:

*H6*: Higher perceived team innovation climate is positively associated with higher graduate student creativity (TIC → PGC).

**Figure 1:**
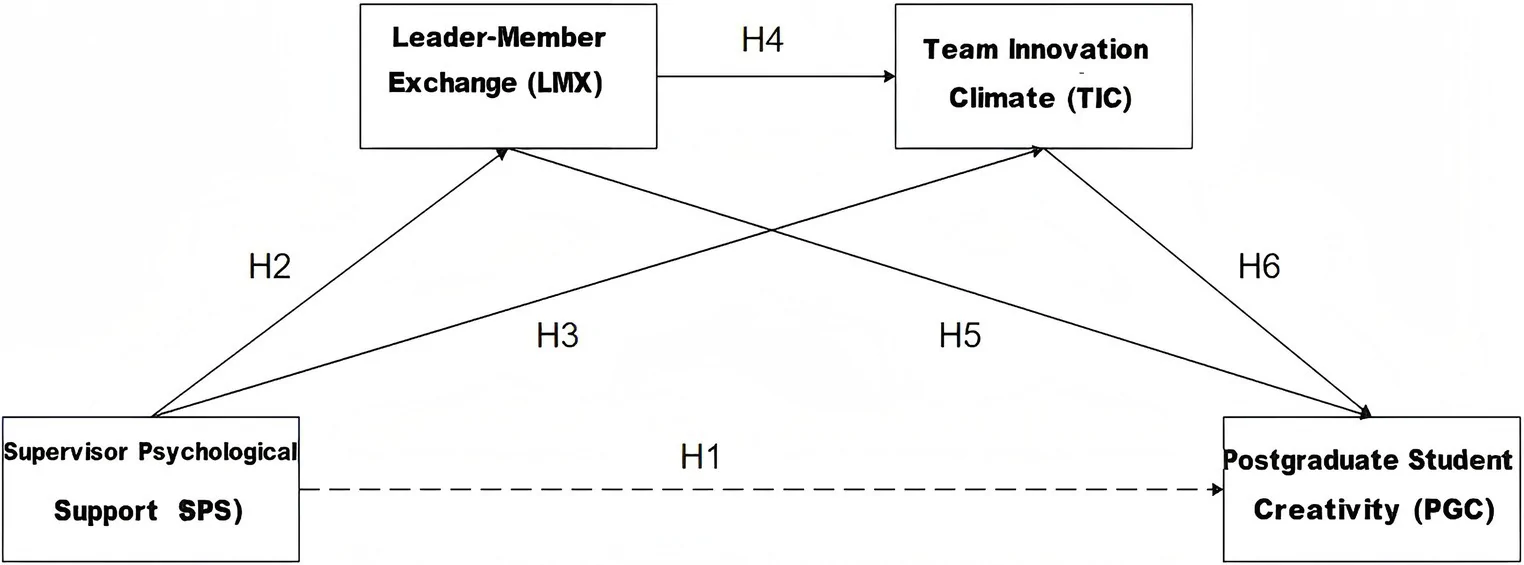
The hypothesized serial mediation model linking supervisory psychological support to graduate student creativity through leader–member exchange and team innovation climate. SPS, supervisory psychological support; LMX, leader–member exchange; TIC, team innovation climate; PGC, graduate student creativity. Solid lines indicate hypothesized significant paths; dashed line represents hypothesized non-significant direct effect.

Within the academic innovation ecosystem, vertical relationships serve as important channels for the transmission of supportive resources (Kwon and Kim, 2020), while individuals’ perception of the team atmosphere operates as the “soil” that nourishes and amplifies the value of such resources (Lee et al., 2014). It is through this interaction of “vertical relationship–perceived atmosphere” that the serial mediation pathway is hypothesized to operate: supervisory psychological support is first hypothesized to cultivate high-quality LMX (H2); high-quality LMX is then hypothesized to shape a positive perceived team innovation climate (H4); and this positive climate is finally hypothesized to be associated with enhanced graduate student creativity (H6). In parallel, LMX (H5) and TIC (H6) are also expected to carry simple mediating effects, while the direct path from SPS to TIC (H3) captures the ambient influence of supervisory support on team-level perceptions. Together, H1 through H6 constitute the hypothesized structural model depicted in Figure 1.

### 2.3 Overview of the hypothesized model

Based on the theoretical integration of self-determination theory, leader–member exchange theory, and social cognitive theory, this study proposes a serial mediation model in which supervisory psychological support is associated with graduate student creativity through six hypothesized pathways (see Figure 1). Specifically:

*H1*: Higher supervisory psychological support is positively associated with higher graduate student creativity (SPS → PGC), as reflected in the total effect. The direct path from SPS to PGC (dashed line in Figure 1) is expected to become non-significant after the mediators are included.

*H2*: Higher supervisory psychological support is positively associated with higher leader–member exchange quality (SPS → LMX).

*H3*: Higher supervisory psychological support is positively associated with higher perceived team innovation climate (SPS → TIC).

*H4*: Higher leader–member exchange quality is positively associated with higher perceived team innovation climate (LMX → TIC).

*H5*: Higher leader–member exchange quality is positively associated with higher graduate student creativity (LMX → PGC).

*H6*: Higher perceived team innovation climate is positively associated with higher graduate student creativity (TIC → PGC).

In addition, the serial indirect pathway (SPS → LMX → TIC → PGC) is expected to make a substantial contribution to the total effect, while the direct path from SPS to PGC (dashed line in Figure 1) is expected to be statistically non-significant after the mediators are included.

### 2.4 Instruments

Data were collected via an online survey battery titled “Graduate Student Creativity and Supervisory Support Survey.” This was not a newly developed single psychometric instrument. Rather, it was a compiled questionnaire consisting of adapted and localized scales corresponding to the four constructs in the proposed model: supervisory psychological support, leader–member exchange quality, team innovation climate, and graduate student creativity. The construct-level structure of the source scales was preserved, while item wording was adjusted to fit the Chinese academic research context and the specific situation of graduate supervision.

All items were rated on 7-point Likert-type scales (1 = Strongly Disagree to 7 = Strongly Agree). The final analytic questionnaire included 28 retained items: 6 items for supervisory psychological support, 7 items for leader–member exchange, 8 items for team innovation climate, and 7 items for graduate student creativity. Each scale was translated and back-translated (English–Chinese–English) following the procedure recommended by Brislin (1986) to ensure semantic accuracy. Scale reliability was assessed using Cronbach’s *α*, with coefficients reported below.

#### 2.4.1 Supervisory psychological support (SPS)

Students’ perceptions of supervisory psychological support were measured using a 6-item scale adapted from the Teacher Autonomy Support Scale (TASS; Black and Deci, 2000). Although the original TASS assesses autonomy support only, Self-Determination Theory posits that optimal functioning requires the synergistic satisfaction of three interrelated basic needs—autonomy, competence, and relatedness (Ryan and Deci, 2017). In the doctoral supervision context, qualitative research by Devos et al. (2015) has established that supervisors’ supportive behaviors systematically encompass autonomy support, structure, and involvement. Following the quantitative precedent of Litalien and Guay (2015), who found that the three need-support dimensions are highly intercorrelated (*r* = 0.75–0.90) and thus computed a general needs support score, we extended the TASS to cover all three needs. Items 1–2 measure autonomy support (e.g., “My supervisor provides me with choices and options in my research”), Items 3–4 measure competence support (e.g., “My supervisor helps me develop the skills needed for my research”), and Items 5–6 measure relatedness support (e.g., “My supervisor shows genuine care and respect for me as a person”). All items were revised to fit the graduate supervision context through translation, back-translation, and expert panel review. Given the small number of items per sub-dimension (*n* = 2) and the theoretical expectation that the three facets operate synergistically in mentoring contexts, the composite SPS score was used as a unidimensional indicator of overall supervisory psychological support. In the present study, the scale demonstrated adequate internal consistency (*α* = 0.86). Higher scores indicate greater perceived supervisory psychological support.

#### 2.4.2 Leader–member exchange (LMX)

The quality of leader–member exchange was assessed using a 7-item scale developed by Liden and Graen (1980), which has been widely validated in higher education contexts (e.g., Shang et al., 2024). The scale captures the quality of the working relationship between supervisor and student, characterized by mutual trust, respect, and reciprocal obligation. Sample items include: “My supervisor understands my research problems and needs,” “My supervisor recognizes my research potential,” and “My working relationship with my supervisor is effective.” Higher scores indicate higher-quality LMX. In the present study, reliability was high (*α* = 0.909), indicating stable measurement properties in the Chinese academic context.

#### 2.4.3 Team innovation climate (TIC)

Perceived team innovation climate was measured using an 8-item scale adapted from the innovation support dimension of the Team Climate Inventory (Anderson and West, 1998), adjusted to fit the characteristics of academic research teams. The scale captures students’ subjective evaluation of the extent to which their research team encourages and supports innovation. The eight retained items cover three aspects: resource collaboration, risk tolerance, and goal consensus. Sample items include: “My research team encourages the integration of diverse knowledge and interdisciplinary methods,” “My research team tolerates failure and does not impose punitive consequences for unsuccessful attempts,” and “Members of my research team jointly recognize that innovation is a core strategic goal.” Higher scores indicate a more positive perceived team innovation climate. In the present study, the scale demonstrated adequate reliability (*α* = 0.888).

#### 2.4.4 Graduate student creativity (PGC)

Graduate student creativity was measured using a 7-item self-rated scale adapted from Zhou and George (2001), capturing the three dimensions of idea generation, practical application, and achievement transformation. The scale assesses students’ self-rated creative behaviors in generating novel and useful ideas in their research activities. Sample items include: “I come up with new and practical ideas to improve my research,” “I search out new technologies, processes, or techniques related to my field,” and “I suggest new ways of performing research tasks.” Higher scores indicate greater self-rated creativity. In the present study, the scale demonstrated adequate reliability (*α* = 0.877) and has shown acceptable validity in the context of Chinese universities.

For all constructs, scales were scored by averaging items so composite scores remained on the original 1–7 scale. By this coding, higher scores on SPS, LMX, TIC, and PGC indicate more perceived supervisory psychological support, higher-quality leader–member exchange, more positive team innovation climate, and greater graduate student creativity, respectively.

### 2.5 Statistical analysis

All quantitative analyses were conducted using IBM SPSS 27.0 and AMOS 29. A two-step analytical strategy was adopted, consistent with recommendations for structural equation modeling: first validating the measurement model, then testing the structural model and mediation hypotheses (Cheung et al., 2024).

#### 2.5.1 Preliminary analyses

Data were inspected for anomalies. Descriptive statistics and Pearson correlations were computed for all key variables (SPS, LMX, TIC, PGC) to understand basic relationships and check for out-of-range values or severe non-normality. Univariate normality was evaluated using the Kolmogorov–Smirnov test, Jarque–Bera test, and Anderson–Darling test. Although these tests are sensitive to large-sample deviations, the absolute values of skewness and kurtosis were examined against conventional thresholds (±3 and ±10, respectively) to determine approximate normality. Multiple collinearity was diagnosed using Pearson correlation coefficients supplemented by variance inflation factor (VIF) and tolerance values.

#### 2.5.2 Common method variance assessment

Given the cross-sectional, self-report nature of the data, potential common method variance (CMV) was evaluated using two complementary procedures following established recommendations in the literature (Podsakoff et al., 2003; Tang and Wen, 2020). First, Harman’s single-factor test was conducted via exploratory factor analysis with all survey items entered unrotated. Second, a confirmatory factor analysis was performed using AMOS 29, in which all measurement items were constrained to load onto a single latent factor. The fit of this single-factor model was compared against the hypothesized four-factor model to assess whether a single common method factor dominated the covariance structure.

#### 2.5.3 Measurement model evaluation

Confirmatory factor analysis (CFA) was conducted on the four core constructs—supervisory psychological support (SPS), leader–member exchange (LMX), team innovation climate (TIC), and graduate student creativity (PGC)—using AMOS 29. The measurement model was evaluated using established criteria (Fornell and Larcker, 1981; Hair et al., 2010). For reflective constructs, internal consistency reliability was assessed using Cronbach’s alpha (*α* > 0.70) and composite reliability (CR > 0.70). Convergent validity was examined through average variance extracted (AVE > 0.50) and standardized factor loadings (*λ* > 0.60). Discriminant validity was assessed using both the Fornell–Larcker criterion and the Heterotrait–Monotrait (HTMT) ratio of correlations (Henseler et al., 2015). We further evaluated discriminant validity by comparing a series of competing measurement models (four-factor, three-factor, two-factor, and single-factor) using CFA.

#### 2.5.4 Structural model testing and mediation analysis

After establishing satisfactory measurement model properties, the structural model representing hypothesized serial mediation relationships was specified. Supervisory psychological support (SPS) was modeled as the exogenous predictor, graduate student creativity (PGC) as the ultimate outcome, and leader–member exchange (LMX) and team innovation climate (TIC) as sequential mediators. Direct paths were included from SPS to each mediator (SPS → LMX, SPS → TIC) and to the outcome (SPS → PGC), as well as paths from LMX to TIC and PGC, and from TIC to PGC.

Mediation analysis was performed using the PROCESS macro (Version 4.2; Hayes, 2013) in SPSS 27.0. All analyses controlled for gender, age, grade level, and academic discipline, and were conducted with 10,000 bootstrap resamples. An indirect effect was deemed statistically significant if its 95% bias-corrected confidence interval did not include zero. The proportion of total effect of SPS on PGC mediated by each specific pathway was calculated to compare relative contributions of simple versus serial mediations.

## 3 Results

### 3.1 Descriptive statistics and preliminary analyses

Table 2 presents descriptive statistics, reliability coefficients, and intercorrelations for all study variables. On average, students reported moderately high levels of supervisory psychological support, leader–member exchange quality, team innovation climate, and graduate student creativity [M(SPS) = 5.46, SD = 0.95; M(LMX) = 5.30, SD = 1.08; M(TIC) = 5.32, SD = 0.92; M(PGC) = 5.15, SD = 0.92], all comfortably above the scale midpoint of 4.00.

**Table 2:** Descriptive statistics, reliabilities, correlations, and convergent validity (*N* = 363).

Variable	M	SD	*α*	CR	AVE	1	2	3	4	VIF
1. SPS	5.46	0.95	0.860	0.89	0.56	(0.748)				2.118
2. LMX	5.30	1.08	0.909	0.91	0.60	0.78***	(0.775)			3.147
3. TIC	5.32	0.92	0.888	0.89	0.54	0.62***	0.63***	(0.735)		2.456
4. PGC	5.15	0.92	0.877	0.88	0.52	0.58***	0.66***	0.66***	(0.721)	2.789

SPS, supervisory psychological support; LMX, leader–member exchange; TIC, team innovation climate; PGC, graduate student creativity. Diagonal values in bold (parentheses) represent square roots of AVE. ***p < 0.001. VIF, variance inflation factor. All VIF values < 5, indicating no significant multicollinearity.

In terms of zero-order correlations, supervisory psychological support had strong positive correlations with LMX (*r* = 0.78, *p* < 0.001), team innovation climate (*r* = 0.62, *p* < 0.001), and graduate student creativity (*r* = 0.58, *p* < 0.001), suggesting that students who perceived greater psychological support from their supervisors tended to report higher-quality exchanges, more positive team climates, and stronger creative outcomes. LMX was also strongly correlated with both team innovation climate (*r* = 0.63, *p* < 0.001) and graduate student creativity (*r* = 0.66, *p* < 0.001). As expected, team innovation climate had a strong positive correlation with graduate student creativity (*r* = 0.66, *p* < 0.001). In contrast, correlations between demographic variables (i.e., gender, age, grade, and academic discipline) and the core study variables were generally weak or non-significant.

Univariate normality tests (Kolmogorov–Smirnov, Jarque–Bera, and Anderson–Darling) were statistically significant for all variables (*p* < 0.05), which is common under large-sample conditions (*N* = 363). However, the absolute values of skewness (range: 0.668–1.400) and kurtosis were well within the conventional thresholds of ±3 and ±10, respectively, indicating that univariate distributions were approximately normal and suitable for parametric analyses including structural equation modeling.

Multiple collinearity was diagnosed using Pearson correlation coefficients supplemented by variance inflation factor (VIF) and tolerance values. The maximum absolute correlation between variables was 0.78 (SPS and LMX), below the empirical threshold of 0.80 for severe multicollinearity. VIF values ranged from 2.118 to 3.147, and tolerance values ranged from 0.318 to 0.472, all within acceptable limits (VIF < 5; tolerance > 0.20). These results indicate that multicollinearity did not threaten the stability of subsequent regression and path estimates.

### 3.2 Measurement model assessment

To assess the convergent validity and internal consistency of the measurement scales, this study conducted confirmatory factor analysis (CFA) on the four core constructs via AMOS 29—supervisory psychological support (SPS), leader–member exchange (LMX), team innovation climate (TIC), and graduate student creativity (PGC)—in accordance with the evaluation criteria proposed by Fornell and Larcker (1981). Results indicated that the standardized factor loadings of all items ranged from 0.69 to 0.84, all of which exceeded the acceptable threshold of 0.6 and were statistically significant (*p* < 0.001), indicating that each item can effectively reflect the latent construct to be measured. In addition, the composite reliability (CR) for each construct was as follows: SPS 0.89, LMX 0.91, TIC 0.89, PGC 0.88, all of which are higher than the ideal critical value of 0.7. The average variance extracted (AVE) of each variable is: SPS 0.56, LMX 0.60, TIC 0.54, PGC 0.52, all of which meet the key determination criterion of convergent validity (AVE > 0.5).

Convergent validity was strongly supported, as average variance extracted (AVE) for all constructs exceeded 0.50 (range: 0.52–0.60), indicating that constructs explain more than half of the variance in their indicators. Additionally, all composite reliability values exceeded corresponding AVE values, further supporting convergent validity.

Discriminant validity was further assessed using the Fornell–Larcker criterion presented in Table 2, wherein the square root of each construct’s AVE (shown on the diagonal in parentheses) exceeded its correlations with other constructs for most variable pairs. Full discriminant validity assessment, including the Heterotrait–Monotrait (HTMT) ratios, is reported in Table 3. Notably, the SPS–LMX correlation (r = 0.78) marginally exceeded the square root of AVE for SPS (0.748); however, given the theoretical proximity between these two constructs in the proposed model, this minor deviation is not uncommon.

**Table 3:** Discriminant validity (HTMT).

Constructs	1	2	3	4
1. SPS	—			
2. LMX	0.886	—		
3. TIC	0.706	0.688	—	
4. PGC	0.663	0.737	0.744	—

SPS, supervisory psychological support; LMX, leader–member exchange; TIC, team innovation climate; PGC, graduate student creativity. All HTMT values <0.90, confirming discriminant validity.

Furthermore, discriminant validity was assessed using the Heterotrait–Monotrait (HTMT) ratio of correlations as a more robust indicator (Henseler et al., 2015). As shown in Table 3, the HTMT value between all construct pairs ranged from 0.663 to 0.886, which is lower than the 0.90 critical threshold recommended by Henseler et al. (2015). This result confirms that the four core constructs in this study—supervisory psychological support (SPS), leader–member exchange (LMX), team innovation climate (TIC), and graduate student creativity (PGC)—all have good discriminant validity and belong to independent conceptual concepts.

To assess potential common method bias, we first conducted Harman’s single-factor test. The results extracted three factors with eigenvalues greater than 1, with the first factor accounting for 44.17% of the total variance, below the critical threshold of 50% (Tang and Wen, 2020), thus indicating that common method bias was unlikely to be a major concern. To further validate this, a confirmatory factor analysis was performed using AMOS 29, in which all measurement items were loaded onto a single factor. The resulting model fit indices were unsatisfactory: *χ*^2^/df = 4.526, CFI = 0.796, TLI = 0.774, RMSEA = 0.101, SRMR = 0.075. The poor fit of this single-factor model provides additional evidence that common method bias does not seriously threaten the validity of our findings.

We further evaluated the discriminant validity of the key constructs by comparing a series of competing measurement models. As summarized in Table 4, the hypothesized four-factor model demonstrated a significantly better fit to the data (*χ*^2^/df = 1.762, RMSEA = 0.046, SRMR = 0.042, TLI = 0.944, CFI = 0.950) than all alternative models (i.e., three-factor, two-factor, and single-factor models). These results support the discriminant validity among the four core variables.

**Table 4:** Results of confirmatory factor analysis.

Factor model	*χ*^2^/df	SRMR	RMSEA	TLI	CFI
Four-factor model	1.762	0.042	0.046	0.944	0.950
Three-factor model (SPS + LMX)	3.283	0.063	0.081	0.844	0.856
Two-factor model (SPS + LMX, TIC + PGC)	3.488	0.071	0.093	0.836	0.843
Single-factor model (SPS + LMX, TIC + PGC)	4.526	0.075	0.101	0.774	0.796

+ indicates factors combined. SPS, supervisory psychological support; LMX, leader–member exchange; TIC, team innovation climate; PGC, graduate student creativity.

### 3.3 Structural model results and hypothesis testing

To test the theoretical model, mediation analysis was performed using the PROCESS macro (Version 4.2; Hayes, 2013), Model 6. All analyses controlled for gender, age, grade level, and academic discipline, and were conducted with 10,000 bootstrap resamples.

**Figure 2:**
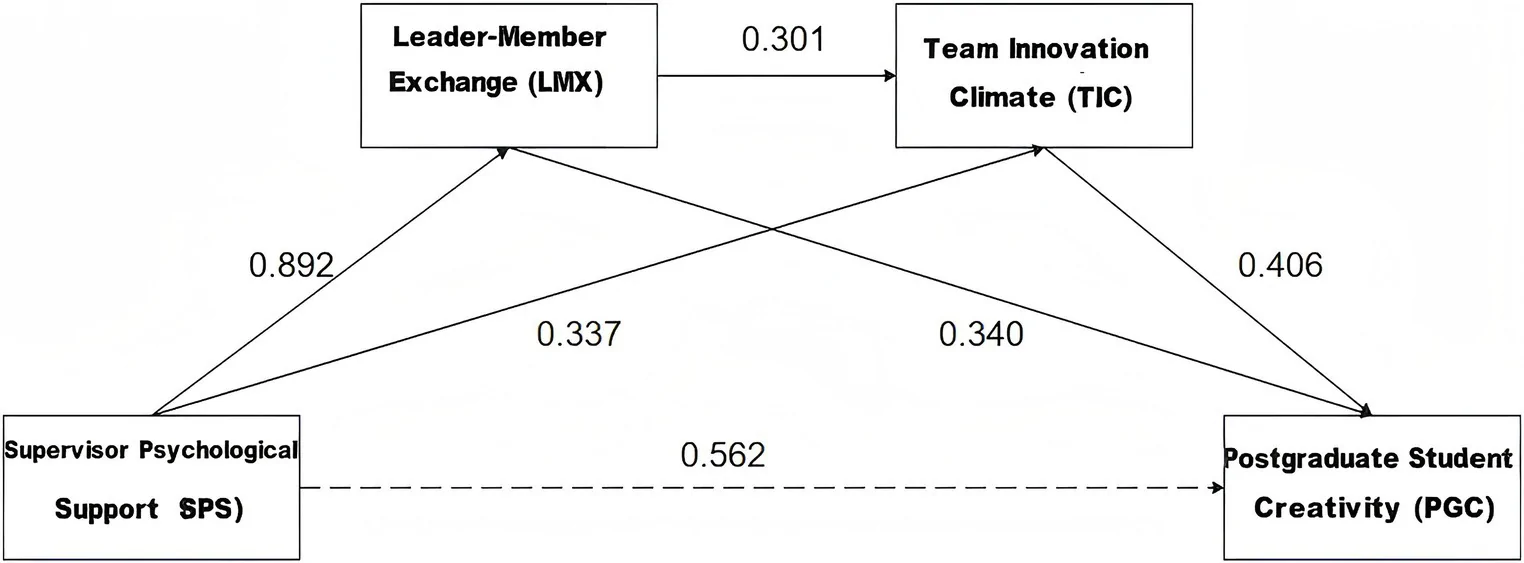
Tested serial mediation model with standardized path coefficients and significance levels. Solid lines indicate significant paths (*p* < 0.001); dashed line represents non-significant direct effect. SPS, supervisory psychological support; LMX, leader–member exchange; TIC, team innovation climate; PGC, graduate student creativity. Path coefficients derived from PROCESS Model 6 with 10,000 bootstrap resamples.

The hypothesized serial mediation model explained substantial variance in graduate student creativity (*R*^2^ = 0.52, *p* < 0.001), indicating that the specified predictors collectively accounted for approximately 52% of the variability in self-rated creative outcomes. Figure 2 presents the tested structural model with standardized path coefficients.

H1 predicted that supervisory psychological support would be positively associated with graduate student creativity (SPS → PGC). Results revealed a significant total effect (*β* = 0.562, SE = 0.048, *p* < 0.001, 95% CI [0.408, 0.707]), thereby supporting the total effect component of H1. However, when LMX and TIC were incorporated as sequential mediators, the direct effect of supervisory psychological support on creativity was no longer significant (*β* = 0.013, SE = 0.059, *p* = 0.826, 95% CI [−0.103, 0.129]). This finding indicates that the association between SPS and PGC is fully mediated by LMX and TIC: the total indirect effect (*β* = 0.549) accounted for the majority of the total statistical association, while the direct path was statistically non-significant.

*H2* predicted that supervisory psychological support would be positively associated with leader–member exchange quality (SPS → LMX). Results revealed a strong positive association (*β* = 0.892, SE = 0.032, *p* < 0.001, 95% CI [0.829, 0.955]), supporting H2. This finding indicates that students perceiving higher levels of supervisory psychological support reported significantly higher-quality mentoring relationships characterized by trust, reciprocity, and mutual obligation.

*H3* predicted that supervisory psychological support would be positively associated with perceived team innovation climate (SPS → TIC). Results revealed that the direct path from SPS to TIC remained significant after controlling for LMX (*β* = 0.337, SE = 0.048, *p* < 0.001, 95% CI [0.243, 0.431]), supporting H3. However, the indirect effect of SPS on TIC via LMX was also significant (*β* = 0.269, BootSE = 0.067, 95% CI [0.149, 0.415]), indicating that LMX functions as a partial mediator in this segment.

*H4* predicted that leader–member exchange quality would be positively associated with perceived team innovation climate (LMX → TIC). Results showed that LMX significantly and positively predicted team innovation climate (*β* = 0.301, SE = 0.041, *p* < 0.001, 95% CI [0.221, 0.381]), supporting H4. This suggests that high-quality supervisor–student relationships generalize into favorable perceptions of the broader team innovation environment.

*H5* predicted that leader–member exchange quality would be positively associated with graduate student creativity (LMX → PGC). Results indicated that, after controlling for SPS and TIC, LMX retained a significant positive association with PGC (*β* = 0.340, SE = 0.052, *p* < 0.001, 95% CI [0.238, 0.442]), supporting H5. This confirms that high-quality relational exchanges provide essential resources and psychological safety that directly facilitate creative outcomes.

*H6* predicted that perceived team innovation climate would be positively associated with graduate student creativity (TIC → PGC). Results showed that, after controlling for SPS and LMX, TIC significantly and positively predicted PGC (*β* = 0.406, SE = 0.058, *p* < 0.001, 95% CI [0.292, 0.520]), supporting H6. This indicates that a supportive team climate independently contributes to graduate student creativity beyond the effects of supervisory support and relational quality.

Taken together, the pattern of results supports a fully mediated statistical architecture: although the total effect of supervisory psychological support on graduate student creativity was substantial (H1 total effect: *β* = 0.562, *p* < 0.001), its direct effect became statistically non-significant once the sequential mediators were included (H1 direct effect: *β* = 0.013, *p* = 0.826). The combined indirect pathways—via LMX alone (H2 → H5), via TIC alone (H3 → H6), and via the serial chain through both mediators (H2 → H4 → H6)—collectively carried the association. This pattern is consistent with the hypothesized model depicted in Figure 1, in which the direct path from SPS to PGC was theorized as non-significant (dashed line) and the creative benefits of supervisory support were expected to unfold sequentially through relational quality and team innovation climate.

### 3.4 Mediation analysis

Table 5 presents the comprehensive decomposition of direct, indirect, and total statistical estimates in the serial mediation model. The total effect of supervisory psychological support on graduate student creativity was substantial and statistically significant (*β* = 0.562, 95% CI [0.408, 0.707], *p* < 0.001), whereas the direct path remained non-significant (*β* = 0.013, 95% CI [−0.103, 0.129], *p* = 0.826). This pattern is consistent with a predominantly indirect statistical model within the tested cross-sectional framework.

**Table 5:** Decomposition of effects: direct, indirect, and total effects of supervisory psychological support on graduate student creativity.

Path	Effect type	Estimate	SE	95% CI Lower	95% CI Upper	Contribution (%)
SPS → PGC	Total effect	0.562	0.048	0.408	0.707	—
SPS → PGC	Direct effect	0.013	0.059	−0.103	0.129	—
SPS → LMX → PGC	Indirect	0.303	—	0.157	0.463	53.91
SPS → TIC → PGC	Indirect	0.137	—	0.069	0.208	24.38
SPS → LMX → TIC → PGC	Indirect	0.109	—	0.054	0.182	19.40

Bootstrap sample = 10,000. SPS, supervisory psychological support; LMX, leader-member exchange; TIC, team innovation climate; PGC, graduate student creativity. CI, confidence interval. SE is omitted for indirect effects because their significance was determined by bias-corrected bootstrap confidence intervals rather than standard errors. Indirect effects were deemed significant if the 95% bias-corrected CI excluded zero. Percentage contributions are calculated relative to the total effect (*β* = 0.562). The direct path point estimate was small and statistically non-significant; therefore, its percentage contribution is not meaningfully interpretable and is omitted from the proportional decomposition.

Examining specific indirect pathways reveals three statistical routes linking supervisory psychological support with graduate student creativity in the specified model.

Pathway 1 (SPS → LMX → PGC; simple mediation via LMX): *β* = 0.303, 95% CI [0.157, 0.463]. This pathway represented the strongest indirect effect, accounting for approximately 53.91% of the total effect. This pattern suggests that the association between supervisory psychological support and graduate student creativity was partly carried through higher-quality leader–member exchange, supporting the mediating role of LMX as specified in H5.

Pathway 2 (SPS → TIC → PGC; simple mediation via TIC): *β* = 0.137, 95% CI [0.069, 0.208]. This pathway accounted for approximately 24.38% of the total effect, indicating that supervisor support was also associated with enhanced creativity through the cultivation of a positive team innovation climate, supporting the mediating role of TIC as specified in H6.

Pathway 3 (SPS → LMX → TIC → PGC; serial mediation via LMX and TIC): *β* = 0.109, 95% CI [0.054, 0.182]. This serial pathway explained approximately 19.40% of the total effect. This represents the theorized statistical sequence in which higher supervisory psychological support is associated with higher LMX quality, which in turn is associated with a more positive team innovation climate, and finally with enhanced graduate student creativity. The magnitude and precision of this estimate support the importance of the sequential psychological pathway in the specified model.

It is critical to emphasize that the statistically non-significant direct effect (*β* = 0.013, *p* = 0.826) does not constitute evidence that the direct pathway is zero in the population. Rather, the wide confidence interval [−0.103, 0.129] indicates that the estimate is imprecise and compatible with both small positive and small negative direct effects. Therefore, the absence of a contribution percentage for the direct path in Table 5 reflects only the non-significant point estimate within the present cross-sectional sample, not a theoretically meaningful absence of direct effect. Throughout this manuscript, terms such as direct effect, indirect effect, and mediation refer to statistical estimates within the specified cross-sectional PROCESS Model 6 framework and should not be interpreted as evidence of causal effects or temporal ordering.

## 4 Discussion

This investigation set out to examine the psychological and social architecture linking supervisory psychological support to graduate student creativity in Chinese higher education, addressing six theoretically derived hypotheses (H1–H6): H1 examined whether supervisory psychological support is directly associated with graduate student creativity; H2, H3, H4, H5, and H6 tested the specific pathways of the proposed mediation architecture—specifically, the associations between SPS and LMX (H2), SPS and TIC (H3), LMX and TIC (H4), LMX and PGC (H5), and TIC and PGC (H6). The empirical results clarify the mechanisms underlying graduate student creativity. In brief, H1 was supported in terms of total effect but not direct effect, while H2 through H6 were all supported, indicating that supervisory psychological support matters primarily through relational and environmental pathways rather than through a direct statistical linkage.

### 4.1 Predominantly indirect association between supervisory support and creativity

One of the most important findings concerns H1, which posited that supervisory psychological support would be positively associated with graduate student creativity. While the total effect was substantial and statistically significant (*β* = 0.562, *p* < 0.001), the direct effect became statistically non-significant (*β* = 0.013, *p* = 0.826, 95% CI [−0.103, 0.129]) once LMX and TIC were included as mediators. This pattern indicates that H1 is supported in terms of total effect but not direct effect, revealing a predominantly indirect statistical architecture. It is important to note that this non-significance does not imply the theoretical absence of a direct effect; rather, the wide confidence interval indicates substantial imprecision, and the estimate is compatible with small yet theoretically meaningful direct effects that the present cross-sectional design and sample size may have been underpowered to detect. At face value, this statistical pattern deviates from the intuitive assumption that supportive, need-satisfying supervisors automatically produce highly creative graduate students. Instead, the findings point to a more nuanced picture: supervisor support alone does not guarantee creative outcomes unless it is first channeled into high-quality exchange relationships and an innovation-friendly team atmosphere. This pattern aligns with emerging evidence that contextual support frequently shows indirect effects through relational and environmental mechanisms, rather than influencing behavior directly (Chen et al., 2009; Meng et al., 2017). Supervisory psychological support may create favorable individual-level conditions, but it is unlikely to be associated with creativity unless students internalize that support as high-quality relational exchange and perceive their broader research team as innovation-friendly. Encouragement and basic need satisfaction may be helpful, but they may not be sufficient to enhance creativity unless students also develop trusting, reciprocal bonds with their supervisors and experience a team climate that tolerates failure and encourages interdisciplinary exploration. In the context of Chinese graduate students—many of whom operate within hierarchical, high-stakes academic systems—the default response even to genuinely supportive supervisors may remain constrained unless relational and environmental channels are activated.

This finding echoes patterns observed in the broader mentoring literature. In hierarchical educational contexts, simply providing intellectual guidance or emotional care does not guarantee innovative outcomes; often, students remain passive or risk-averse unless they feel embedded in high-quality exchange relationships that provide both psychological safety and tangible resource access (Zhao et al., 2019; Shang et al., 2024). Analogously, the present results suggest that awareness of supervisory support is a necessary but insufficient condition for creative outcomes. The sufficient conditions appear to be the student’s perception of high-quality LMX and an innovation-conducive team atmosphere.

### 4.2 Serial mediation through relational quality and team innovation climate

The serial mediation pattern can be understood through the joint lens of SDT, LMX theory, and social cognitive theory, and is underpinned by the empirical support for H2 through H6. Specifically, H2 (SPS → LMX: *β* = 0.892, *p* < 0.001) confirmed that supervisory psychological support is strongly associated with higher-quality mentoring relationships. H4 (LMX → TIC: *β* = 0.301, *p* < 0.001) established that these relational qualities generalize into more positive team innovation climates. H5 (LMX → PGC: *β* = 0.340, *p* < 0.001) and H6 (TIC → PGC: *β* = 0.406, *p* < 0.001) demonstrated that both relational quality and team climate independently facilitate creative outcomes. Rather than acting as a direct trigger, supervisory psychological support thus sets off a chain reaction: it is first associated with higher-quality mentoring relationships (H2), which then relate to more positive team innovation climates (H4), and subsequently to greater self-rated creativity (H5, H6). This stepwise pattern fits SDT’s argument that need satisfaction enhances intrinsic motivation primarily when embedded in meaningful social contexts (Ryan and Deci, 2017). Thus, support does not operate in a vacuum; its creative benefits seem to unfold through the sequential activation of relational and environmental conditions.

Similar indirect architectures have been documented in related fields. For instance, transformational leadership has been shown to influence teacher burnout primarily via organizational support and resilience (Karacabey et al., 2025), and leadership effects on organizational innovation are frequently transmitted through relational quality followed by climate perceptions (Hülsheger et al., 2009). The present study extends this emerging pattern to graduate education: the sequence from contextual support (H2) → relational resource (H4) → environmental perception (TIC) → creative outcome (H5, H6) appears to hold even when the outcome is graduate student creativity and the context is hierarchical academic mentoring. This suggests that the “support–relationship–climate–outcome” chain may represent a broader psychological regularity across hierarchical social settings, rather than being unique to classrooms or organizations.

The strong positive link observed from supervisory psychological support to LMX (H2: *β* = 0.892) is particularly informative. It suggests that a substantial part of LMX quality is associated with students’ perception that their basic psychological needs are being met by the supervisor. This has practical implications for mentor training. Traditional approaches often treat need support and relationship building as separate goals—for example, by using workshops to teach feedback techniques while using separate retreats to build rapport. The present results are more consistent with an integrated approach: supervisors who systematically satisfy students’ needs for autonomy, competence, and relatedness may simultaneously cultivate the high-quality exchange relationships that serve as the primary conduit for creativity.

Furthermore, H3 (SPS → TIC: *β* = 0.337, *p* < 0.001) revealed that supervisory psychological support retains a significant direct association with team innovation climate even after controlling for LMX, indicating that LMX functions as a partial mediator in this segment. This finding is theoretically important because it suggests that supervisors influence team climate through both relational and ambient channels—pathways not fully captured by the dyadic relationship alone. This partial mediation pattern reinforces the theoretical distinction between vertical (supervisor–student) and horizontal (team-level) mechanisms, suggesting that both levels warrant attention in academic interventions.

### 4.3 Cultural specificity: high power distance and collectivism

High power distance and relational investment. First, the high power distance characteristic of the Chinese academic system profoundly shapes the underlying logic of supervisor–student interactions. In such contexts, supervisors’ control over critical academic resources (e.g., research funding, publication opportunities, graduation approval) renders high-quality LMX an essential pathway for students to acquire the means necessary for innovation (Anand et al., 2011; Zheng et al., 2018). In the Chinese academic situation, supervisory psychological support—such as autonomy encouragement and competence feedback—may be interpreted not merely as the satisfaction of individual psychological needs, but also as a signal of relational investment, reflecting the supervisor’s intention to establish emotional connection and inclusiveness at the relational level (Chen et al., 2009). This interpretation is consistent with the core view of social exchange theory (Blau, 1986): in high power distance structures, supportive behavior is first perceived as a sign of relationship quality, which in turn is associated with reciprocal engagement. According to the cross-cultural meta-analysis of Rockstuhl et al. (2012), the formation of LMX in China has distinct affective orientation (humanity/relationship) characteristics, which contrasts sharply with the mainstream contribution-orientation logic of the West. Therefore, in the hierarchical interaction between supervisors and students, building a high-quality mentoring relationship has become a key prerequisite for students to obtain the resources and psychological security required for innovative activities (Farh et al., 1998; Chen et al., 2009). The strong support for H2 (SPS → LMX) and H5 (LMX → PGC) empirically corroborates this relational investment logic: students must first enter the supervisor’s “in-group” through high-quality LMX to gain access to the resources and psychological safety that enable creative risk-taking.

Collectivism and team environment shaping. Second, the collectivism and long-term orientation of Chinese culture further reinforce the above “relationship-first” pathway and lead to team-level environment cultivation (Ouyang et al., 2025). Collectivist norms emphasize harmony, interdependence, and group goals (Hofstede, 2001; Triandis, 1995), which lead supervisory support behaviors to focus not only on individual development but more on maintaining team harmony through cultivating close vertical relationships, thereby being associated with a shared innovation climate. This process reflects signaling mechanisms (Connelly et al., 2011; Erdogan and Bauer, 2014): when supervisors invest in high-quality LMX through psychological support, this behavior signals a commitment to innovation to the team, prompting students to generalize individual-level support perceptions to team-level innovation climate (Schneider and Reichers, 1983). The significant paths H4 (LMX → TIC) and H3 (SPS → TIC) indicate that this generalization operates through both relational radiation and direct supervisory modeling. Under this cultural script, the stimulation of individual creativity is more often viewed as a natural derivative of high-quality relationships and positive team environments rather than achieved through direct activation of individual motivation alone (Zhou and Su, 2010).

Taken together, high power distance and collectivism jointly explain why supervisory psychological support in the Chinese academic context is channeled primarily through relational and environmental pathways (H2–H6) rather than directly stimulating individual creative output (H1 direct effect). Whether this culturally embedded pattern generalizes to educational systems with lower power distance or more individualistic norms remains an open empirical question.

### 4.4 Theoretical implications for cross-cultural research

The following discussion is speculative and theory-driven, as the present sample was drawn exclusively from mainland China. These propositions require direct empirical testing in Western contexts before they can be treated as established cultural differences. The observed mediation pattern may differ in Western academic contexts. In Western contexts such as the U.S. and the U.K., power distance is significantly lower, and individualist norms are dominant, reflecting a cultural emphasis on the independent self (Hofstede, 2001; Markus and Kitayama, 1991; Triandis, 1995). Supervisor–student relationships tend to be more egalitarian, and creativity is traditionally framed as an individual-level attribute driven by personal intrinsic motivation. Support might be associated with creativity more directly by meeting basic needs (Ryan and Deci, 2017), without necessarily going through relational quality first.

From “social embeddedness” to “individual drive”: differences in mediation mechanism weights. It is important to emphasize that this difference is not an absolute opposition but rather a relative difference in the weights of mediation mechanisms. Western studies have similarly confirmed that LMX mediates the association between leadership behaviors and creativity (e.g., Tierney et al., 1999; Gerstner and Day, 1997). A comprehensive meta-analytic review by Lee et al. (2020) further substantiated that leadership–creativity linkages are frequently transmitted through relational and motivational mechanisms, converging with the present finding that LMX serves as a primary mediator. However, in low power distance, individualistic cultures, students may directly seek innovative resources through institutional channels or individual initiative regardless of LMX quality; conversely, in Chinese high power distance contexts, high-quality LMX appears to be a necessary condition for resource acquisition and psychological safety, thereby explaining the absence of a significant direct effect in the present sample (H1 direct effect: *β* = 0.013, *p* = 0.826).

In Western individualistic cultures, due to the emphasis on the independent self and intrinsic motivation, the association between supervisor support and creativity is expected to be more direct and less dependent on relationship building as a prerequisite. Western cross-cultural creativity research traditionally adopts an “individual-centered approach,” viewing creativity as the result of internal individual drive (Zhou and Su, 2010), and therefore typically reports direct effects or partial mediation effects of psychological support on creativity. In such contexts, H1 might yield a significant direct effect, and the relative contributions of H5 (LMX → PGC) and H6 (TIC → PGC) may be smaller than those observed in the present Chinese sample.

In contrast, in Chinese collectivist culture, creativity has social embeddedness—individuals must first obtain “in-group status” through relational networks (LMX) to gain access to key academic resources (Farh et al., 1998; Liden and Graen, 1980) and establish psychological safety through reciprocity mechanisms based on social exchange (Blau, 1986), before personal motivation can be translated into creative output. This may explain why the direct effect was not statistically significant in the present sample (H1), whereas significant direct effects or partial mediation effects are typically reported in Western contexts. Nevertheless, this difference may also reflect measurement variability or sampling fluctuations, and should be interpreted cautiously pending longitudinal replication.

### 4.5 Practical implications

The predominantly indirect model revealed in this study—wherein H2 through H6 collectively carry the effect of supervisory psychological support on creativity, while H1 direct effect is statistically non-significant—suggests that in Chinese academic contexts, supervisory psychological support must be transformed into student creativity through the serial pathway of “relationship building” and “environment cultivation.” The results of this study have three layers of inspiration for the practice of postgraduate training:

First, at the mentor level, colleges and universities should promote the paradigm shift from transaction-based “task support” to relational “relationship investment.” In view of the strong support for H2 (SPS → LMX: *β* = 0.892) and its downstream effects on H4, H5, and the serial pathway, mentor training programs should not only focus on scientific research skills guidance or general psychological care, but also systematically cultivate supervisors’ relationship-building ability—including establishing emotional connections, providing non-instructive support (such as focusing on students’ career development, not just focusing on the output of papers), and accumulating “relationship capital” through high-frequency and high-quality one-on-one interaction. Specifically, structured workshops should help supervisors realize that in China’s high-power culture, students’ perception of support will be cognitively filtered through relationship quality. If only the support frequency is increased without improving the quality of LMX, the marginal return may decrease, as the present data indicate that LMX functions as the primary “social converter” (H2, H5) between psychological need satisfaction and creative output.

Second, at the level of team building, it is necessary to build an innovative ecosystem with relationships as the link. As H3 (SPS → TIC) and H4 (LMX → TIC) indicate that team innovation climate is shaped by both direct supervisory signals and relational generalization, academic team intervention should adopt a two-track strategy: on the one hand, transmit the signal of failure tolerance through the supervisor’s demonstration behavior; on the other hand, establish a horizontal peer support mechanism to transform vertical supervisor–student relationship resources into team sharing resources. Specifically, laboratories or research teams can set up an “innovation safety circle” to conduct regular failed case studies, spreading the individualized psychological security provided by supervisors into a collective atmosphere so as to realize the indirect statistical association between LMX and creativity.

Third, at the level of institutional design, “relationship quality” indicators should be included in the evaluation system of colleges and universities. At present, the evaluation of postgraduate training mostly focuses on the scientific research output of supervisors or the output of students’ papers, ignoring the process-oriented relationship quality. Given that H5 and H6 identify LMX and TIC as the primary conduits for creativity—together explaining 97.69% of the total association between SPS and PGC—it is recommended that colleges and universities introduce students’ evaluation dimensions of relationship quality (such as trust degree, emotional support reach) in the selection and assessment of supervisors, and establish an early warning and intervention mechanism for low LMX relationships. This practice is especially applicable to China’s academic system: when supervisors hold the key power in the allocation of academic resources, the institutional guarantee can effectively avoid the inhibition of students’ creativity by low-quality exchange relations.

## 5 Research limitations and future directions

While this study provides novel insights into mechanisms of graduate student creativity, several limitations warrant acknowledgment. First, the cross-sectional design limits the ability to draw firm causal conclusions. The proposed ordering of supervisory psychological support, leader–member exchange, team innovation climate, and graduate student creativity is grounded in Self-Determination Theory, LMX theory, and social cognitive theory, but the data cannot establish temporal precedence. Some associations may be reciprocal or bidirectional. For instance, highly creative students may elicit more supervisory attention and resources, which would strengthen the quality of LMX; similarly, improvements in the team innovation climate may also prompt supervisors to provide greater support. Longitudinal designs following students over time, cross-lagged models, or intervention studies would be needed to test causal direction more rigorously.

Second, the focus was on perceived rather than objective creativity. The PGC scale captured students’ self-rated creative ideation, practical application, and achievement transformation, but it did not incorporate supervisor evaluations, peer nominations, or objective innovation indicators such as patents or published papers. Future research is encouraged to use multi-source assessment to distinguish between perceptual bias and objective creative facts.

Third, context was limited to full-time graduate students at multiple comprehensive universities in mainland China. Caution is needed in generalizing to other educational settings. Graduate students in more academically elite institutions, professional degree programs, or non-Chinese cultural contexts may exhibit different mediation patterns. Future studies could test the model in different countries or at different education levels to examine its robustness.

Fourth, data relied solely on self-report questionnaires. These were appropriate for internal states such as LMX quality and climate perceptions, but there is room to enrich evidence with more objective measures. Behavioral observations (such as coding the frequency of creative idea proposals in team meetings) or multi-informant ratings (supervisor-rated creativity, peer-rated LMX) would help validate whether the observed statistical associations manifest in observable behaviors.

Finally, this study did not distinguish between master’s and doctoral students, nor did it examine potential boundary conditions. There are significant differences between the two groups in terms of the duration of the supervisor–student relationship, creativity standards, and the degree of academic socialization, which may moderate the mediation pathways. Future research should adopt stratified random sampling and explore the moderating effects of degree level, disciplinary field, or supervisor seniority on the mediation mechanism. Additionally, although team innovation climate was conceptualized as a team-level construct, the analysis was conducted at the individual level due to insufficient team-level sample sizes; future research should employ multilevel modeling to properly account for the nested structure of students within research teams (Hox et al., 2017).

## 6 Conclusion

This study tested a theoretically specified serial mediation model linking supervisory psychological support to graduate student creativity through six hypothesized pathways (H1–H6). The empirical results provide a coherent narrative: H2, H3, H4, H5, and H6 were all supported, revealing that supervisory psychological support operates through relational quality (LMX) and team innovation climate (TIC) to foster creativity. H1 was supported in terms of total effect (*β* = 0.562, *p* < 0.001) but not direct effect (*β* = 0.013, *p* = 0.826), indicating a predominantly indirect statistical pattern wherein the association between support and creativity is carried almost entirely by the proposed mediators. Because this study was cross-sectional, these pathways should be interpreted as theory-guided statistical patterns rather than as evidence of causal mechanisms or temporal ordering.

Graduate students in China are often described as lacking creativity or being overly dependent on their supervisors. This characterization is both partially accurate and incomplete. It is accurate in the sense that the direct statistical pathway from supervisory support to creativity was not statistically distinguishable from zero once LMX and TIC were accounted for (H1 direct effect: *β* = 0.013, *p* = 0.826). It is incomplete because the findings suggest that creativity is associated with identifiable social-psychological conditions—high-quality mentoring relationships (H2, H5) and supportive team climates (H3, H4, H6)—rather than fixed dispositional passivity. Within the limits of a cross-sectional design, the results indicate that these conditions may be meaningfully addressed through support that builds relational quality and cultivates team innovation climate.

The central finding qualifies a common pedagogical intuition: perceived supervisory psychological support did not show a significant direct statistical pathway to graduate student creativity once LMX and TIC were included in the model (H1). Supportive and need-satisfying supervisors remain important, but the results suggest that support is most relevant when students convert it into a stronger appraisal of their mentoring relationship quality (H2: *β* = 0.892) and team environment (H3: *β* = 0.337; H4: *β* = 0.301). When that relational and environmental appraisal is low, even genuinely supportive supervision may leave creative potential untapped. This is not a pessimistic conclusion; rather, it points to the importance of precise, relationship-oriented intervention.

The serial mediation chain quantified here situates the primary psychological bottleneck at leader–member exchange rather than at team climate alone. Supervisory psychological support was positively associated with LMX (H2: *β* = 0.892), LMX was positively associated with team innovation climate (H4: *β* = 0.301), and team innovation climate was positively associated with creativity (H6: *β* = 0.406). Meanwhile, both LMX (H5: *β* = 0.340) and TIC (H6: *β* = 0.406) retained significant direct associations with creativity when included simultaneously. Programs that address graduate student creativity only through individual skill training or generic motivational encouragement, without cultivating genuine mentoring relationships and team-level innovation norms, may be targeting downstream symptoms rather than central social pathways. The findings therefore suggest that supervisor training and graduate education curricula in Chinese institutions should sequence relationship-building and climate-shaping opportunities before, or alongside, individual creativity techniques.

The predominantly indirect pattern identified in the present data warrants careful attention in subsequent research. The absence of a significant direct effect (H1), within a cross-sectional self-report framework, aligns with social-embeddedness dynamics characteristic of high power-distance educational environments. Whether this reflects the necessity of Guanxi-based resource access, the collective nature of academic evaluation in China, or something more specific to the hierarchical structure of graduate supervision remains an open empirical question. The current data can identify the statistical pattern with precision; explaining its mechanism will require qualitative follow-up and longitudinal designs that the present study was not structured to provide.

Certain limitations bear directly on the scope of these conclusions. The cross-sectional design precludes causal inference, and restriction to self-reported measures raises questions about common method variance despite the statistical controls employed. Whether the mechanisms specified in H1–H6 hold in graduate institutions outside mainland China, or in systems where the social meaning of supervision differs substantially, is empirically open. The exclusive reliance on student self-report also means that participants’ described creativity may not map perfectly onto observable innovative behavior, a gap that future observational or multi-informant work would be well positioned to close.

Despite these boundaries, the findings suggest that graduate student creativity in Chinese higher education is not randomly distributed but systematically patterned around modifiable relational and environmental conditions. The model accounted for 52% of the variance in creativity, implying that peer networks, disciplinary traditions, and institutional policies may explain the remaining share. Even so, the support–LMX–climate chain—empirically validated through H2–H6—offers a sizeable and actionable starting point for educators seeking to improve graduate innovation through evidence-based mentoring and team interventions.

## Data Availability

The raw data supporting the conclusions of this article can be found at: https://doi.org/10.6084/m9.figshare.31889938.
