# Supplementary material for: Supervisory psychological support and graduate student creativity: a serial mediation model
Source: Front Psychol. 2026 Aug 12;17:1857534. doi: 10.3389/fpsyg.2026.1857534 (PMC13506485; doi:10.3389/fpsyg.2026.1857534)
Supplement: Supplementary file 1 [file Supplementary_file_1.DOCX]

**Questionnaire of Core Study Variables**

**Note:** This document contains all measurement items used in the above manuscript. All scales are adapted from well-established, validated instruments in prior academic literature, with contextual adjustments for the Chinese graduate education context. All items except demographic questions are rated on a 7-point Likert scale (1 = Strongly Disagree, 7 = Strongly Agree).

**I. Demographic Items**

# 1. Your gender

2. Your age range
3. What year of graduate study are you currently in?
4. Does your major belong to liberal arts or science?

**II. Supervisor Psychological Support Items**

# 5. My supervisor seriously considers my different research ideas

# 6. My supervisor's feedback has significantly improved my analytical abilities

# 7. My supervisor recommends learning resources (e.g., literature/tools) to address my weaknesses

# 8. My supervisor provides specific suggestions for improvement regarding my research progress

# 9. I feel fully academically respected within the team

# 10. My supervisor actively cares about my academic progress and stress levels

**III. Supervisor-Student Relationship Items**

# 11. My supervisor actively helps me solve key problems in my research

# 12. My communication with my supervisor is smooth and our collaboration is efficient

# 13. I have sufficient confidence in my supervisor; even when my supervisor is not present, I would support his/her academic decisions

# 14. My supervisor considers my different ideas

# 15. My supervisor and I have a high degree of tacit understanding in academic collaboration

# 16. My supervisor understands my problems and needs

**IV. Team Innovation Climate Items**

# 17. The team regularly organizes brainstorming sessions to discuss new ideas

# 18. Team members are willing to provide resources (e.g., data, equipment) to support the practice of new ideas

# 19. Team members collaborate to advance the implementation of new ideas

# 20. Team members can freely express different opinions, even if they represent a minority view

# 21. Team members can understand and accept each other

# 22. Team members actively share research-related information

# 23. Team members have a clear consensus on research goals

# 24. The team encourages trying risky research methods without criticism even if they fail

**V. Creativity Level Items**

# 25. I often propose research hypotheses or theoretical frameworks that differ from existing literature

# 26. My research methods integrate multidisciplinary tools

# 27. My innovative solutions have significantly improved research efficiency

# 28. I can verify innovative hypotheses through rigorous empirical processes

# 29. My innovative solutions are feasible under existing resource conditions

# 30. My innovative ideas have been recognized or cited by others

**Research Ethics**

This study strictly adheres to academic research ethics standards. Prior to the questionnaire survey, all participants were fully informed of the research purpose, content, and procedures. Participation was emphasized as entirely voluntary, and participants were informed that they could withdraw from the survey at any time without any adverse consequences. All data were anonymized during the research process, with strict protection of participants' personal privacy and identity information. The data will be used solely for academic research analysis and will not be disclosed to any third parties.

**Data Visibility (Data Transparency)**

This study will follow academic data openness standards. After cleaning and anonymizing the raw questionnaire data, the data will be stored on encrypted servers for a retention period of no less than 5 years. Upon completion of the thesis, the anonymized dataset, data analysis code, and detailed workflow documentation will be uploaded to public academic platforms to ensure that research results can be replicated and verified by peers. Meanwhile, the thesis appendix will provide detailed explanations of variable coding rules, missing value handling methods, and sample descriptive statistics to ensure transparency and traceability of the data processing and analysis procedures.
